# Vascular Collagen Type-IV in Hypertension and Cerebral Small Vessel Disease

**DOI:** 10.1161/STROKEAHA.122.037761

**Published:** 2022-10-07

**Authors:** Apoorva A. Kumar, Natalie Yeo, Max Whittaker, Priya Attra, Thomas R. Barrick, Leslie R. Bridges, Dennis W. Dickson, Margaret M. Esiri, Chad W. Farris, Delyth Graham, Wen Lang Lin, Daniel N. Meijles, Anthony C. Pereira, Gregory Perry, Douglas L. Rosene, Anan B. Shtaya, Tom Van Agtmael, Giovanna Zamboni, Atticus H. Hainsworth

**Affiliations:** Molecular and Clinical Sciences Research Institute, St George’s University of London, United Kingdom (A.A.K., N.Y., M.W., P.A., T.R.B., L.R.B., D.N.M., A.C.P., G.P., A.B.S., A.H.H.).; Neurology (A.A.K., A.C.P., A.H.H.), St George’s University Hospitals NHS Foundation Trust, London, United Kingdom.; Cellular Pathology (L.R.B.), St George’s University Hospitals NHS Foundation Trust, London, United Kingdom.; Department of Neuroscience, Mayo Clinic, Jacksonville, FL (D.W.D., W.L.L.).; Nuffield Department of Clinical Neurosciences, Oxford University, United Kingdom (M.M.E., G.Z.).; Department of Anatomy and Neurobiology, Boston University School of Medicine, MA (C.W.F., D.L.R.).; Institute of Cardiovascular and Medical Sciences, University of Glasgow, United Kingdom (D.G., T.V.A.).; Dipartimento di Scienze Biomediche, Metaboliche e Neuroscienze, Università di Modena e Reggio Emilia, Italy (G.Z.).

**Keywords:** Alzheimer disease, animals, collagen, hypertension, linear models

## Abstract

Cerebral small vessel disease (SVD) is common in older people and causes lacunar stroke and vascular cognitive impairment. Risk factors include old age, hypertension and variants in the genes *COL4A1/COL4A2* encoding collagen alpha-1(IV) and alpha-2(IV), here termed collagen-IV, which are core components of the basement membrane. We tested the hypothesis that increased vascular collagen-IV associates with clinical hypertension and with SVD in older persons and with chronic hypertension in young and aged primates and genetically hypertensive rats.

**Methods:**

We quantified vascular collagen-IV immunolabeling in small arteries in a cohort of older persons with minimal Alzheimer pathology (N=52; 21F/31M, age 82.8±6.95 years). We also studied archive tissue from young (age range 6.2–8.3 years) and older (17.0–22.7 years) primates (*M mulatta*) and compared chronically hypertensive animals (18 months aortic stenosis) with normotensives. We also compared genetically hypertensive and normotensive rats (aged 10–12 months).

**Results:**

Collagen-IV immunolabeling in cerebral small arteries of older persons was negatively associated with radiological SVD severity (ρ: −0.427, *P*=0.005) but was not related to history of hypertension. General linear models confirmed the negative association of lower collagen-IV with radiological SVD (*P*<0.017), including age as a covariate and either clinical hypertension (*P*<0.030) or neuropathological SVD diagnosis (*P*<0.022) as fixed factors. Reduced vascular collagen-IV was accompanied by accumulation of fibrillar collagens (types I and III) as indicated by immunogold electron microscopy. In young and aged primates, brain collagen-IV was elevated in older normotensive relative to young normotensive animals (*P*=0.029) but was not associated with hypertension. Genetically hypertensive rats did not differ from normotensive rats in terms of arterial collagen-IV.

**Conclusions:**

Our cross-species data provide novel insight into sporadic SVD pathogenesis, supporting insufficient (rather than excessive) arterial collagen-IV in SVD, accompanied by matrix remodeling with elevated fibrillar collagen deposition. They also indicate that hypertension, a major risk factor for SVD, does not act by causing accumulation of brain vascular collagen-IV.

Cerebral small vessel disease (SVD) is common in older people and is a major cause of lacunar stroke, deep intracerebral hemorrhage, and vascular cognitive impairment.^1–3^ Older age and hypertension are the main risk factors for SVD.^1^ Neuroimaging features that are reported as manifestations of SVD include lacunar infarcts, diffuse white matter lesions (WML) and subcortical microhemorrhages.^1,4^ The recognized vascular pathology of SVD is concentric, fibrotic thickening of the walls of deep penetrating arteries (outer diameter ≈20–200 microns).^2,3^

Nonfibrillar collagen type IV, consisting of alpha chains 1 and 2 (α1[IV], α2[IV]) are a major structural component of the basement membrane (BM) in all blood vessels.^5,6^ Triple helical trimeric collagen-IV molecules, consisting of 2 α1(IV) and 1 α2(IV) chains, are secreted and form a strong but flexible lattice-style network within the vascular BM.^5,6^

Human genetic data consistently implicate collagen-IV in SVD.^6^ Monogenic forms of SVD are caused by coding mutations in *COL4A1* or *COL4A2*^6–8^ which are adjacent genes encoding α1(IV) and α2(IV) collagens. These familial mutations lead to BM dysfunction, manifesting with early onset SVD of variable severity.^5–7^ Recent analysis also identified rare coding variants occurring in patients with sporadic intracerebral hemorrhage.^9^ In addition to monogenic SVDs, the *COL4A1-COL4A2* locus is associated with the much more-common sporadic SVD. Large genome-wide association studies associate common intronic single-nucleotide polymorphisms (SNPs) in *COL4A1-COL4A2* with SVD phenotypes, including lacunar stroke,^10,11^ subcortical hemorrhages,^10,11^ and diffuse WMLs.^10,12^ As these SNPs are noncoding, their effects on phenotype likely reflect either excessive or insufficient levels of vascular collagen-IV.

The biological relation between vascular collagen-IV and sporadic SVD remains poorly understood. Based on the reported excessive vascular collagen-IV levels in some rare monogenic forms of SVD,^13,14^ we hypothesized that in sporadic SVD, *COL4A1-COL4A2*-mediated risk is due to excessive vascular collagen-IV. As vessel wall fibrosis is a cardinal feature of hypertensive vascular disease, with earlier studies reporting increased collagen deposition,^15–17^ we further hypothesized an association of increased vascular collagen-IV with hypertension. To address this, we used a cross-species approach including human brain tissue, macaques with aortic stenosis,^18^ and genetically hypertensive rats.^19^ Contrary to our hypothesis, our data suggest that vascular collagen-IV is negatively associated with sporadic SVD and that expression levels are influenced by aging but not by hypertension.

## Methods

Further experimental details are given in the Supplemental Material.

This work adhered to STROBE (Strengthening the Reporting of Observational Studies in Epidemiology) guidelines for human studies and ARRIVE (Animal Research: Reporting of In Vivo Experiments) guidelines for animal studies. The data that support the findings of this study are available via the corresponding author upon reasonable request.

### Research Involving Biological Material and Data From Human Participants

Human tissue samples were supplied by Oxford Brain Bank (REC approval#15/SC/0639). Written informed consent was received from participants or their next-of-kin prior to inclusion in the study. Ethical approval for use of human brain tissue in this study was provided by National Research Ethics Service (East Midlands-Derby research ethics committee, Ref#12/EM/0028). The study was performed in accordance with the ethical standards as laid down in the 1964 Declaration of Helsinki and its later amendments.

### Research Involving Animals

Nonhuman primate studies were approved by the Boston University Institutional Animal Care and Use Committee. Animals were maintained in the Laboratory Animal Science Center of Boston University, which is accredited by the Association for the Assessment and Accreditation of Laboratory Animal Care. All animals were treated with strict accordance to the standards of the NIH Guide for the Care and Use of Laboratory Animals. Rat studies were approved by the University of Glasgow Ethical Review Panel and complied with the Animals (Scientific Procedures) Act 1986.

### Human Brain Tissue

A well-defined cohort of older individuals who had minimal Alzheimer disease pathology (Braak neurofibrillary tangle stage 0–II) were studied, for details see our previous report.^20^ Demographic data are in Table 1. Frontal and parietal cortical tissue blocks containing subcortical white matter were examined. As a radiological measure of SVD severity, the severity of diffuse WMLs was rated on a categorical scale (WML score, range: 0–3) based on previously published scales.^21,22^ Neuropathological diagnosis of SVD was defined by as in our previous studies,^2,20^ using standard pathological criteria, including small vessel wall thickening, widened perivascular spaces, and parenchymal changes considered to result from SVD.^2^ Similar brain areas were also examined from brains of younger adults without evidence of neurological or psychiatric disease (n=4: 1F/3M; mean [SD] age: 33.3 [9.7], range 20–41 years).

**Table 1.**
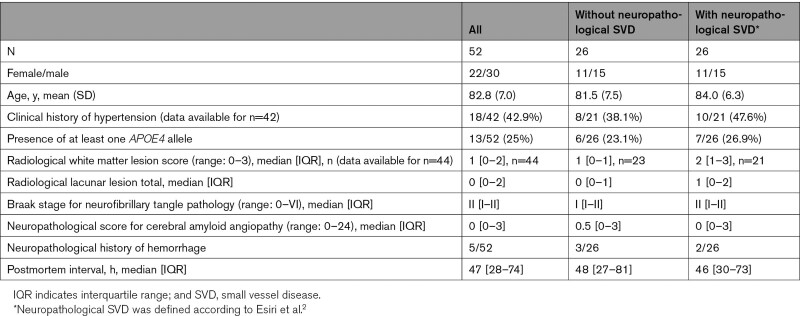
Demographic Data for the Cohort of Aged Human Brains

Formalin-fixed paraffin-embedded sections were immunohistochemically labeled as described previously^20^ using a monoclonal antibody selective for α1(IV) and α2(IV) collagens (clone COL-94, mouse IgG_1_, Sigma-Aldrich, Poole, United Kingdom).

Immunolabeled sections were viewed on a Nikon Eclipse Ni-E upright microscope under 20× or 40× objective lens. All vessels of arterial appearance within subcortical white matter in the size range 40 to 150 μm least outer diameter were digitally sampled in TIFF format. Collagen-IV positive area fraction (%) within each vessel wall was calculated as area fraction =100×(collagen-IV positive vessel wall area/total vessel wall area). Mean area fraction was calculated from all vessels meeting the inclusion criteria for each case. Sclerotic index was computed as 1.0−(inner diameter/outer diameter). Harvesting of TIFF files and all image analyses were performed blind to clinical data.

### Immuno-Electron Microscopy

Thin sections of human brain were immunogold labeled for collagens I, III, and IV (Rockland Immunochemicals, Gilbertsville, PA) as in our previous work.^23^

### Primate Model

Adult male *M* *mulatta* were randomized either to undergo aortic narrowing to induce chronic severe hypertension^18^ or to remain unoperated (normotensive controls). Chronic hypertension was produced by surgically coarcting the aorta, details of which we have described.^24^ Following recovery from surgery, animals were hypertensive for at least 18 months before brain magnetic resonance imaging, euthanasia, and immunohistochemical labeling for collagen-IV (mouse monoclonal antibody, clone COL-94).

### Hypertensive Rat Model

Male rats aged 10 to 12 months from inbred colonies of genetically hypertensive animals (stroke-prone spontaneously hypertensive rat) and age-matched normotensive animals (Wistar-Kyoto parent strain) were compared as in our previous report.^19^ Formalin-fixed paraffin-embedded sections were immunohistochemically labeled as in our previous work^19^ for collagen-IV (rabbit polyclonal IgG #600-401-106, Rockland Immunochemicals, Gilbertsville, PA).

### Statistics

Statistical testing was performed in SPSS (v.26). Continuous variables were assessed for normal distribution ((Kolmogorov-Smirnov test). Between-group differences were tested using 2-tailed Student *t* tests or Mann-Whitney *U* tests as appropriate. Univariate general linear models were used to investigate the relations between extent of collagen-IV immunolabeling (as dependent variable) with presence/absence of clinical history of hypertension and neuropathological SVD as fixed factors, and with age at death, radiological SVD severity (WML score), and postmortem interval (PMI) as covariates. No post hoc corrections were applied. *P*<0.05 was considered significant.

## Results

### Cerebral Vascular Collagen-IV in Older People

**Figure 1.**
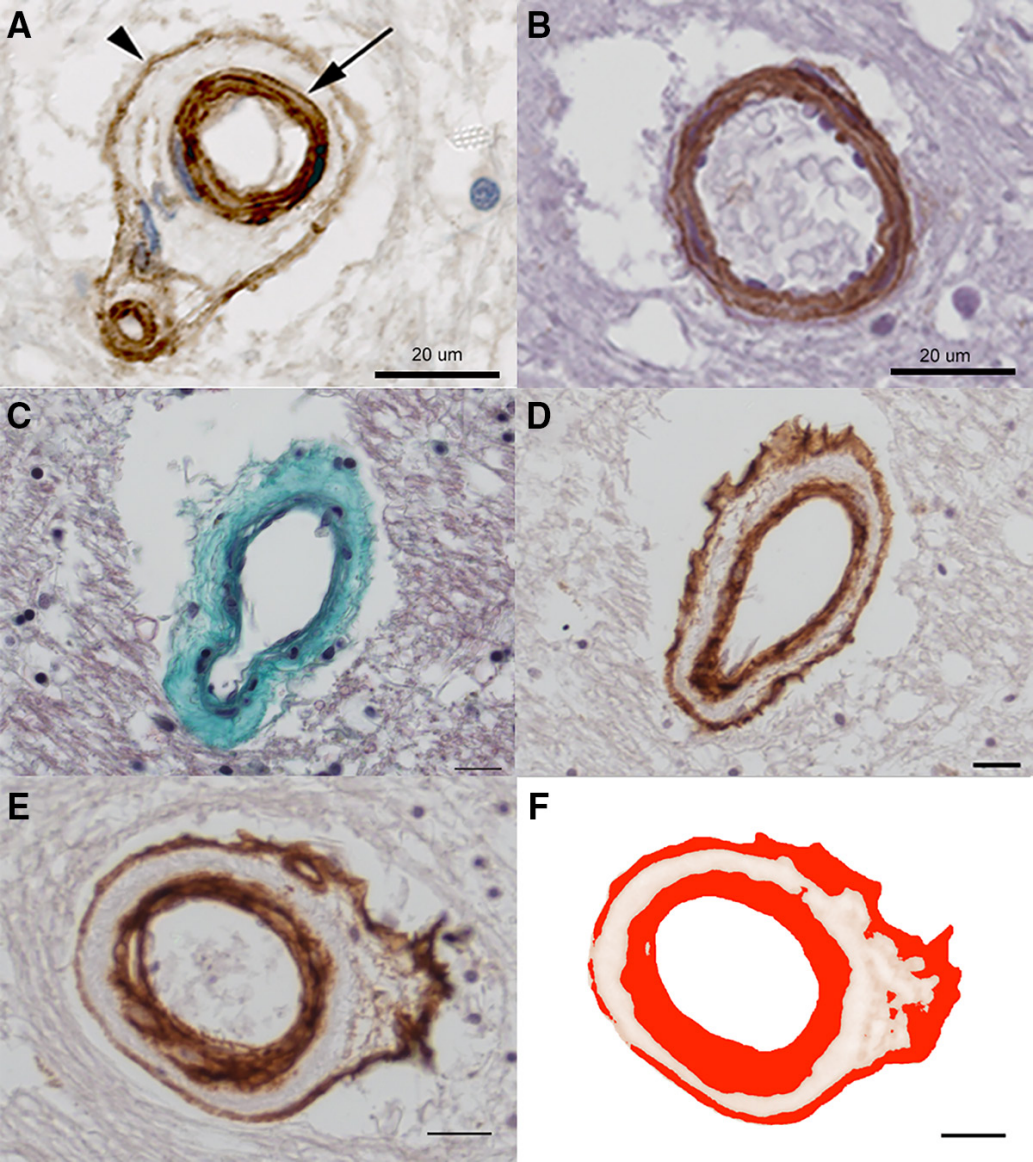
**Vascular collagen-IV in small arteries of human brain. A, C–F** are from older people, whereas **B** is from a young adult (male, age 20). **A**, Small penetrating artery in subcortical white matter of aged human brain shows multilaminar collagen-IV (brown) in the subendothelial basement membrane (arrow) and an outer layer of adventitial collagen-IV (arrowhead). Hematoxylin chromatin counterstain (blue). **B**, A comparable artery in a young adult exhibits a qualitatively different collagen-IV pattern, with a thinner vessel wall spanned entirely by layers of collagen-IV positive matrix. **C** and **D**, Neighboring sections from an older person, treated with Masson trichrome stain (**C**) or collagen-IV immunolabeling (**D**). A small artery shows acellular, fibrotic thickening of the medial layer, stained green with the trichrome stain (**C**). Collagen-IV (brown) is clearly present in the subendothelial basement membrane (BM) and adventitial layers but not the fibrotic medial area. **E** and **F**, Example of automated measurement of the vascular collagen-IV labeled area fraction (AF). Pixels that are labeled within the vessel wall for collagen-IV (brown, **E**) are detected by an automated algorithm (red, **F**). Scale bars: 20 μm.

We examined neuropathological tissue from a well-defined cohort of older individuals with minimal AD pathology (N=52, mean [SD] age: 82.8 [6.95], range 65–99 years; clinical details in Table 1). In small penetrating arteries, the subendothelial BM was universally collagen-IV positive, often multilayered, and separated from an adventitial layer of collagen-IV that ensheathed the vessel, giving an appearance of concentric collagen-IV positive rings (Figure 1A). A qualitatively different pattern of vascular collagen-IV was observed in similar arteries of younger adults (n=4, age 33.3 [9.7] years) where BM and adventitial collagen-IV were continuous (Figure 1B). Confocal imaging confirmed the separation of subendothelial and adventitial layers of collagen-IV immunoreactivity in arteries of older adults (Figure S1). Standard histological techniques indicated that the unlabeled zone between the 2 layers of collagen-IV contained poorly cellular fibrotic tissue (see trichrome staining, Figure 1C and 1D).

**Figure 2.**
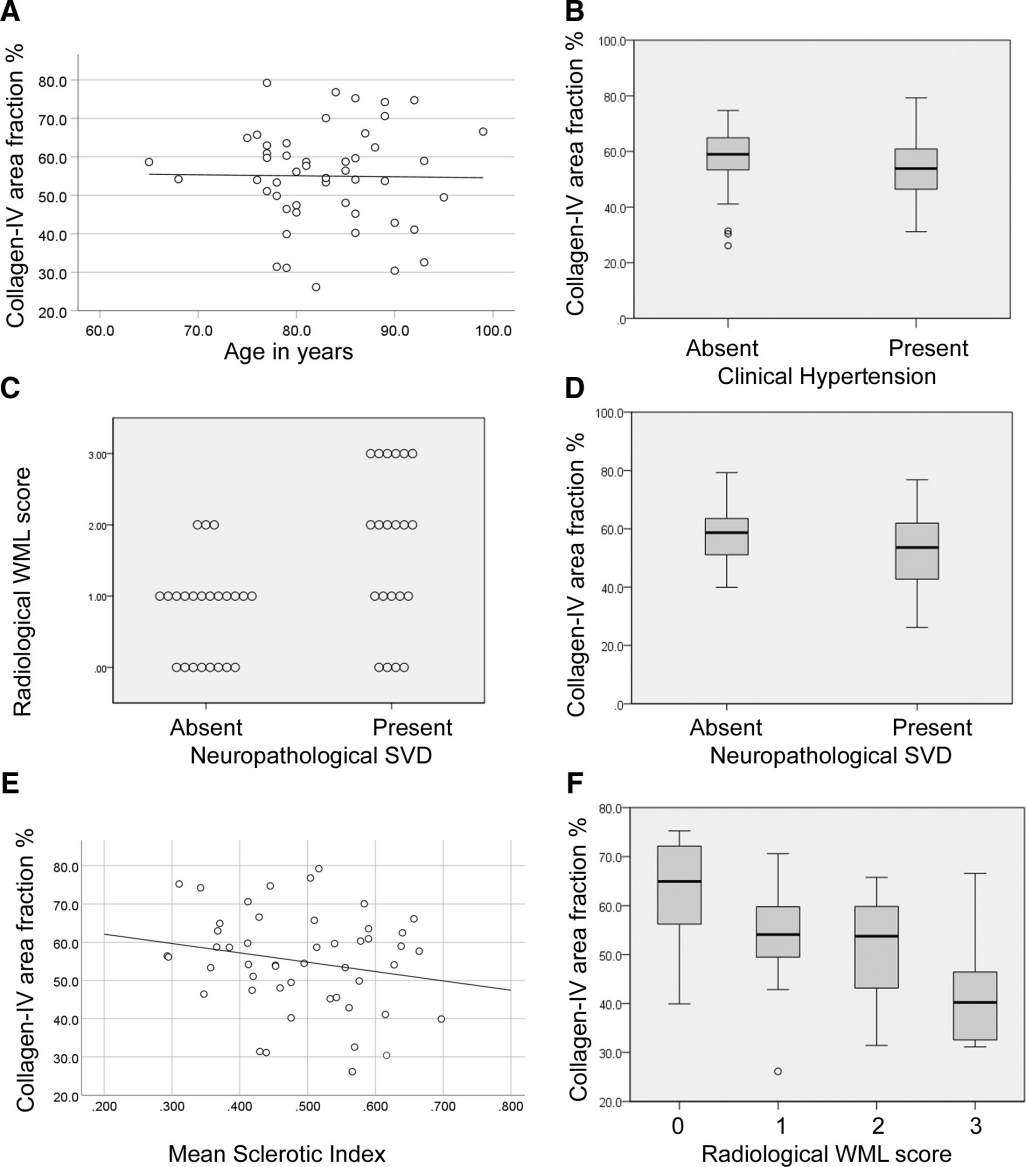
**Vascular collagen-IV in older people.** The extent of vascular collagen-IV within small penetrating arteries was quantified as the collagen-IV immunolabeled area fraction (AF) of the vessel wall. **A**, Average collagen-IV in relation to age at death. Straight line shows least-squares best fit. **B**, Collagen-IV in relation to the absence or presence of clinical history of hypertension. **C**, Radiological white matter lesion (WML) score in relation to the absence or presence of neuropathological diagnosis of small vessel disease (SVD). **D**, Collagen-IV in relation to absence or presence of neuropathological SVD diagnosis. **E**, Collagen-IV in relation to average sclerotic index for small arteries. Straight line shows least-squares best fit. **F**, Collagen-IV in relation to radiological WML score. In **A**, **C**, and **E**, each symbol represents a different person. Box-whisker plots show median, interquartile range (IQR), and full range, with outliers that depart more than 3×IQR. Data from 52 older people with minimal Alzheimer pathology.

We next quantified the collagen-IV positive area fraction of the vessel wall for small arteries (Figure 1E and 1F). We tested whether collagen-IV immunolabeling was associated with: age at death, clinical history of hypertension, PMI, neuropathological diagnosis of SVD,^2^ mean sclerotic index, or WML score as a radiological index for SVD severity (Supplemental Methods). Only PMI departed from normality (Kolmogorov-Smirnov statistic=0.129, *P*=0.033). Across the cohort of older people, vascular collagen-IV was not significantly associated with age (Pearson R: −0.014, *P*=0.913; Figure 2A) or with PMI (Spearman ρ: 0.159, *P*=0.281). Females and males differed in terms of age at death (86.6 [5.75] versus 79.8 [6.49] years, respectively; *P*<0.001, Student *t* test) but not in terms of vascular collagen-IV (*P*=0.164). Clinical history of hypertension trended higher prevalence among male subjects than females (*P*=0.0653, Fisher exact test). See Table S1 for demographic data of male and female subjects.

Vascular collagen-IV did not significantly differ between persons with or without a documented history of hypertension (*P*=0.734, Student *t* test, Figure 2B). Those with neuropathological diagnosis of SVD had significantly higher radiological WML score than those without (*P*=0.006, Mann-Whitney *U* test; Figure 2C). Persons with neuropathological SVD diagnosis exhibited a trend for lower vascular collagen-IV, relative to those without neuropathological SVD (*P*=0.082, Student *t* test, Figure 2D) but collagen-IV was not significantly associated with sclerotic index, a histological measure of arterial wall thickening (Pearson R: −0.203, *P*=0.162; Figure 2E). Vascular collagen-IV was significantly negatively associated with WML score, a radiological marker of SVD (Spearman ρ: −0.427, *P*=0.005, Figure 2F).

We then used general linear models to test further whether vascular collagen-IV was related to clinical history of hypertension, neuropathological SVD diagnosis, age at death, radiological WML score or PMI. Results from 4 models of increasing complexity are given in Table 2. Models with collagen-IV as dependent variable and with radiological WML score as covariate were significant when either hypertension or neuropathological SVD were included as fixed factors (*P*=0.010, 0.009, respectively; Model 1; Table 2), although not with both factors included. When PMI (Model 2), or age at death (Model 3), or both (Model 4) were added as covariates, the models remained significant with either hypertension (*P*≤0.030) or neuropathological SVD (*P*≤0.022) included as a fixed factor, but not with both (Table 2). In all 4 models, vascular collagen-IV was negatively associated with radiological WML score (*P*≤0.017) but not with age or PMI (Table 2). These results support a negative association between WML score and vascular collagen-IV that is not driven by hypertension, chronological age (among older persons), or by PMI.

**Table 2.**
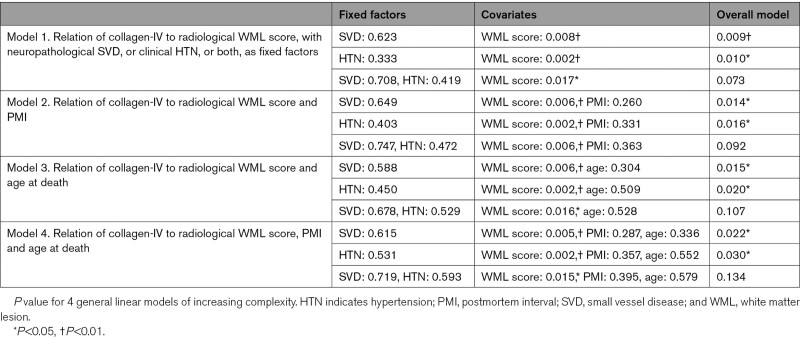
Univariate General Linear Models for Collagen-IV

### Electron Microscopy Shows Matrix Remodeling in Human Small Arteries

**Figure 3.**
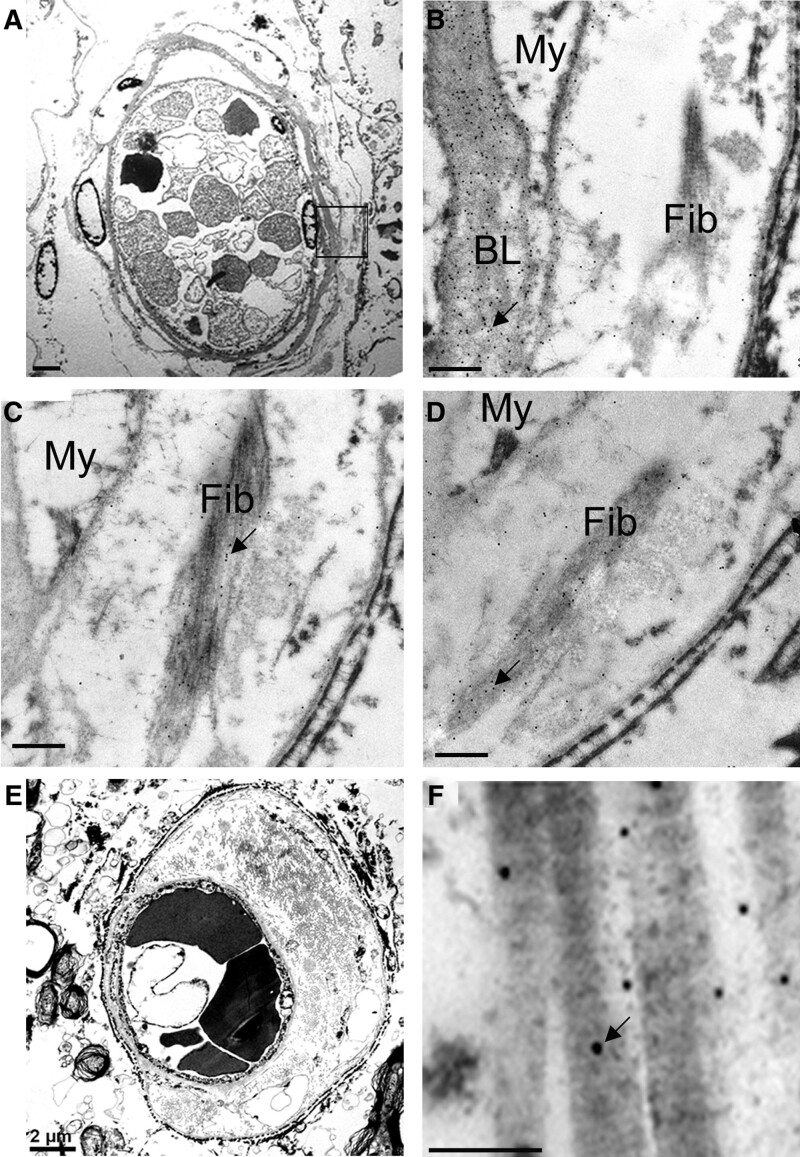
**Immuno-electron microscopy of collagens I, III, and IV in small vessel disease (SVD).** Transmission electron micrographs of small arteries in white matter from older persons with SVD. **A–D**, Semi-serial sections of a small arteriole, shown at low magnification in **A** and at higher magnification in **B–D** with immunogold labeling for collagen-IV (**B**), collagen-I (**C**), or collagen-III (**D**). The region shown at higher magnification in **B–D** is marked in **A** (box). Arrows show examples of immunogold particles. In **B**, collagen-IV labeling shows numerous gold particles over the homogenous basal laminae (BL), behind the endothelium. Mural collagen fibrils (Fib) and myocytes (My) are unlabeled. **C**, Collagen-I, in contrast to collagen-IV, is mainly localized to the collagen fibrils. **D**, Collagen-III also labels the banded collagen fibrils. A few gold particles are found over the basal laminae. **E** and **F**, Another small artery with severe fibrosis. Low magnification view (**E**) shows heavy deposits of fibrillar collagens in the vessel wall. In higher magnification, these are heavily labeled for collagen-I (**F**). Scale bars: 2 μm (**A** and **E**), 0.5 μm (**B–D**), and 0.2 μm (**F**).

Immunogold transmission electron microscopy was used to explore subcellular labeling for collagen-IV and fibrillar collagens (types I and III) in small arteries of people with SVD. Collagen-IV was abundant in mural extracellular matrix, forming BM in the subendothelial area and around medial myocytes (Figure 3B). Collagen-IV was not seen within endothelial cells or myocytes (Figure 3B). Collagen fibrils were seen in the fibrotic artery wall (Figure 3). These fibrillar bundles were positive for collagen-I and collagen-III but not for collagen-IV (Figure 3B through 3F). Standard immunohistochemical labeling confirmed collagen-I throughout the vessel wall (Figure S2). Collagen-III was also consistently detected but with a less-extensive labeling pattern through the vessel wall (Figure S2).

### Vascular Collagen-IV in Chronically Hypertensive Animal Models

**Figure 4.**
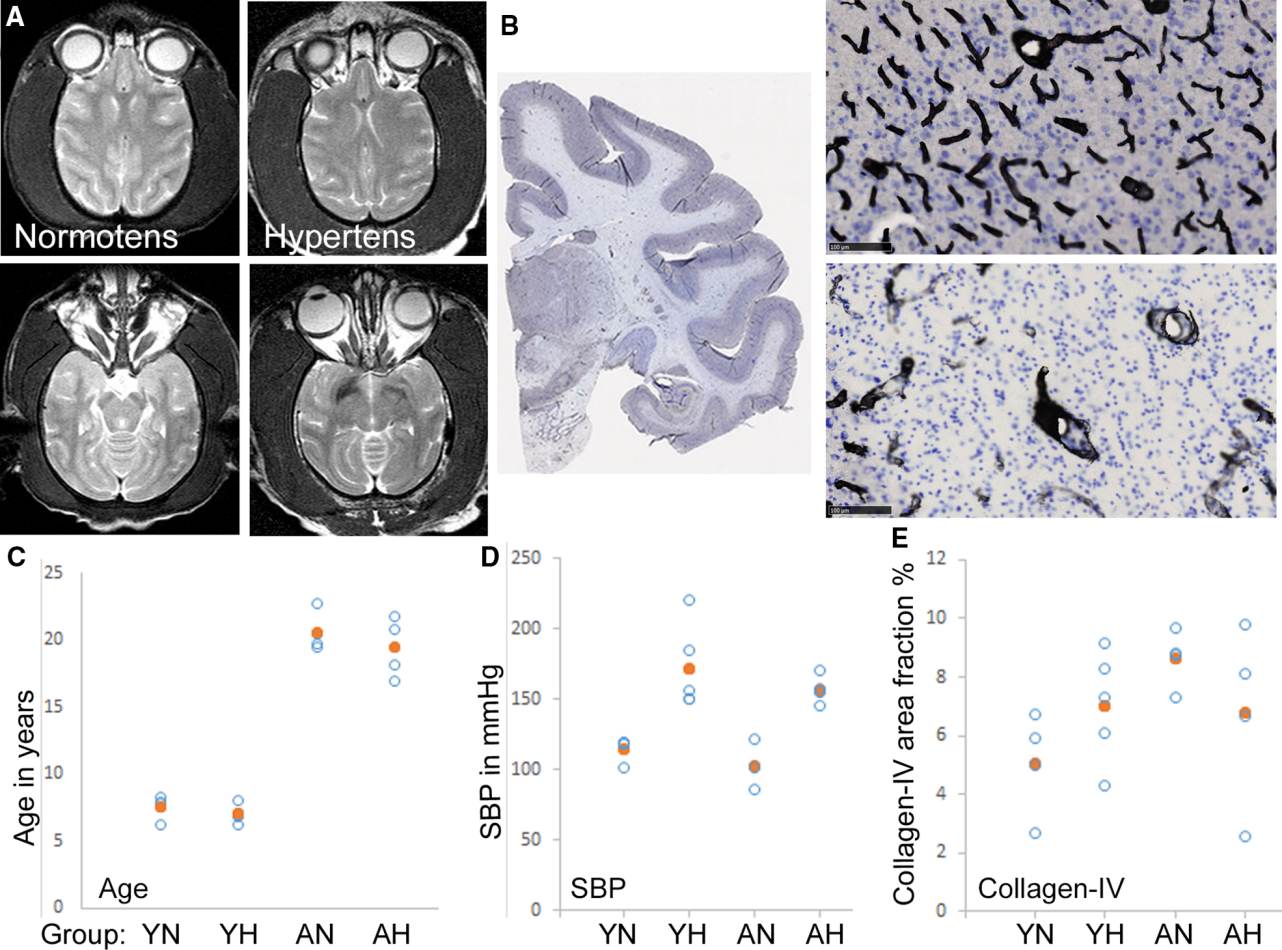
**Collagen-IV in hypertensive primate brains. A**, T2-weighted magnetic resonance imaging (MRI) scans of a normotensive (**left**) and chronically hypertensive primates (**right**) show no white matter lesion (WML), at 2 different horizontal planes. The normotensive animal was aged 7.5 y when scanned. The hypertensive animal was aged 16.9 y and had been hypertensive for 2.6 y when scanned. **B**, Collagen-IV immunolabeled (black) cerebral cortex of primate, with hematoxylin chromatin counterstain (blue). A low magnification view is shown, with examples of collagen-IV labeled vessels in gray matter (**upper**) and subcortical white matter (**lower**). Scale bars: 100 µm. **C–E**, Scatter plots showing age at death (**C**), systolic blood pressure (SBP; **D**), and extent of white matter collagen-IV labeling (**E**) in young normotensive and hypertensive (YN and YH) and aged normotensive and hypertensive primates (AN and AH). Open symbols represent individual animals, filled symbols show the group mean. Data from 17 animals.

To test prospectively whether age and hypertension modulate vascular collagen-IV, we examined the area density of brain collagen-IV immunoreactivity in young and aged macaques, in a surgical model of chronic hypertension (Figure 4). Animals were hypertensive for 29.3 (4.3) months (mean [SD]; range: 18.7–33.1 months). Hypertensive animals were compared with normotensive controls, in young adults aged 7.3 (0.79) years at sacrifice (range: 6.2–8.3 years, n=9) and older adults, aged 20.0 (1.9) years (range: 17.0–22.7 years, n=8). SBP was significantly higher in young hypertensive primates, relative to young controls, and in aged hypertensive primates, relative to aged controls (*P*=0.0107, 0.0197, respectively; Student *t* test; Figure 4). No animals exhibited WML on T2-weighted magnetic resonance imaging scans 26.8 (2.7) months (range: 23.8–31.2 months) after surgery (Figure 4A). Immunolabeling for collagen-IV (Figure 4B) revealed significantly more extensive staining in white matter of aged normotensive relative to young normotensive adults (*P*=0.029, Mann-Whitney *U* test) but did not differ between aged hypertensive adults and young hypertensive adults (*P*=0.999), or between hypertensive and normotensive animals within the young adult (*P*=0.190) or aged adult groups (*P*=0.486; Figure 4C through 4E).

Effects of chronic hypertension on vascular collagen-IV were also interrogated in the stroke-prone spontaneously hypertensive rat, a well-established model of hypertension-associated cerebrovascular disease. Chronically hypertensive male stroke-prone spontaneously hypertensive rat (mean [SD] age: 344 [34] days) from an inbred colony were compared with age-matched male rats (age 329 [13] days) from the sister colony of normotensive Wistar-Kyoto parent strain. Leptomeningeal arteries exhibited fibrotic wall thickening in hypertensive relative to normotensive rats (Figure S3). Mean sclerotic index of leptomeningeal arteries was higher in hypertensive relative to normotensive rats (*P*=0.038, Mann-Whitney *U* test; Figure S3E) but the extent of vascular collagen-IV immunolabeling in these vessels was not significantly different (*P*=0.762, Mann-Whitney *U* test; Figure S3F).

## Discussion

The main findings of this report are as follows. Contrary to our hypothesis, the extent of vascular collagen-IV was not related to history of hypertension in older humans (Figure 2; Table 2), or in chronically hypertensive macaques (Figure 4) or in genetically hypertensive rats (Figure S3). In older humans, the extent of vascular collagen-IV had a strong negative association with WML score, a radiological marker of SVD severity (Figure 2F). This relationship between radiological SVD severity (ante mortem) and a microscopic measure of vascular collagen-IV immunolabeling was preserved across 4 general linear models (Table 2). Ultrastructural data confirmed the lack of vascular collagen-IV accumulation in SVD and supported accumulation of fibrillar collagens I and III, as part of SVD arterial pathology (Figure 3).

The possible association of hypertension with vascular collagen-IV has been addressed previously, in smaller cohorts of human tissue (N=14–27).^15–17,25^ In accord with our data, putaminal microvascular collagen-IV did not differ between older people with or without hypertension.^25^ By contrast, others reported BM thickening in hypertensive people, claimed to be due to laminin and collagen-IV (although these data were observational, not quantitative).^17^ There are several previous studies in experimental animals. Some support increased vascular collagen-IV with high blood pressure (in contrast to our findings),^26,27^ while others do not.^28,29^ Our cross-species analysis highlights the need to discriminate between fibrillar collagens and BM collagen (type IV).

The 2 animal models were used to test a molecular hypothesis (that hypertension increases collagen-IV expression) rather than to mimic the human disease of sporadic SVD. For this hypothesis-testing study, we used only male animals to avoid the variations of the female sex hormones in the female sex cycle. Both species were studied at a higher age range than is usually seen in experimental studies, although neither could be considered old. Male macaques reach sexual maturity at about 5 years. Their aging differs from human aging by ≈3-fold. Monkeys surviving over age 30 are rare, incidence of cognitive impairment increasing steadily from 20 to 30 years of age.^30^ For healthy male laboratory rats, normal life expectancy is 2 to 3.5 years. As male stroke-prone spontaneously hypertensive rats have high mortality prior to 10 months of age, survival bias may be present in our rat data. Even so, the rats studied here (age 10–12 months) exhibited substantial arterial thickening and fibrosis (Figure S3 and our previous article).^19^ Survival bias is unlikely either in our primate data (no unexpected mortality was seen in this cohort prior to planned sacrifice) or in human data, as sporadic SVD is rarely fatal.

Older people exhibited a qualitatively different pattern of arterial collagen-IV from that in young adults, with separation of subendothelial BM from a distinct adventitial sheath in older adults (Figure 1A and 1B). These findings agree with previous immunohistochemical reports^15^ and also with ultrastructural data^31,32^ where a reduplication of the subendothelial BM is noted in older humans and animals, in electron microscopy.^32^ Similarly, aged normotensive primates had more extensive brain collagen-IV relative to young primates (Figure 4E). These findings support the concept that aging is associated with increased and altered collagen-IV distribution in small arteries.

The negative association between arterial collagen-IV and radiological SVD severity in older people is contrary to our hypothesis of excessive collagen-IV in sporadic SVD. Instead, it suggests that insufficient collagen-IV in brain arteries associates with SVD or at least with a radiological SVD profile. We speculate that this may be related to loss of vascular myocytes, which is a salient feature of sporadic SVD. Association is not causality, and it may be that radiological WML and depleted arterial collagen-IV both result from a common cause, possibly an upstream aspect of arterial pathology. Neuropathological diagnosis of SVD trended a negative association with vascular collagen-IV (*P*=0.08). Alternatively, we speculate that heightened vessel fragility or permeability, due to insufficient BM collagen-IV, may have a causal role in sporadic SVD. Non-sense mutations in *COL4A1* can lead to familial SVD, which strongly supports insufficient collagen-IV as a cause of vascular fragility.^33^

The association of arterial collagen-IV with radiological SVD provides insight into genetic risk of sporadic SVD.^10,11^ In human genome-wide association studies data, SVD phenotypes (diffuse WML, lacunar infarcts, and deep hemorrhage) were associated with intronic SNPs in the *COL4A1/COL4A2* locus.^10–12^ As these SNPs do not change the amino acid sequence, their likely impact is on the degree, or cellular location, of *COL4A1/COL4A2* expression. Each BM collagen-IV protomer consists of 2 α1(IV) and 1 α2(IV) subunits.^5,6^ Hence, modifying the abundance of either the α1(IV) or the α2(IV) isoforms can affect BM integrity. Moreover, *COL4A1* and *COL4A2* are organized in a head-to-head orientation and the 2 genes use a single, bidirectional promoter. Thus, intronic SNPs that are related to SVD risk may influence expression of both genes. Based on previous reports of excessive vascular collagen-IV in a rare monogenic SVD,^13,14^ our initial supposition was that increased expression of α1(IV) and α2(IV), contributing to arterial wall fibrosis, might be causal in sporadic SVD. Instead, our findings support insufficient mural collagen-IV as a pathogenic factor in sporadic SVD.

Fibrosis is a well-established pathological process, seen in many chronic diseases, usually with an inflammatory component.^34^ Drivers include inflammatory cytokines (IL [interleukin]-1β, IL-6, and TNF [tumor necrosis factor]-α), growth factors (TGF [transforming growth factor]-β1 and PDGF [platelet-derived growth factor]), and matrix polysaccharides (hyaluronan and chondroitin). We speculate that sporadic SVD encompasses a fibrotic component, in the vessel wall of small arteries supplying deep brain tissue. Central to fibrosis are myofibroblasts, which secrete abundant extracellular matrix components, notably fibrillar type I and type III collagens.^34^ Our data suggest that type IV collagen is not part of the fibrotic process in SVD.

Ultrastructural data confirm that excessive collagen-IV is not a feature of small artery fibrosis in sporadic SVD. Collagen-IV was absent from the acellular, hyaline material of fibrotic small arteries of people with SVD. This acellular material was positive for fibrillar collagens I and III, consistent with previous studies in familial forms of SVD^13,35^ and in severe, sporadic SVD.^15,16^ Fibril-forming collagens cause tensile stiffness due to their arrangement as highly orientated fibrillar structures.^34,36^ The underlying pathogenesis of fibrotic protein deposition in cerebral small arteries remains unknown, although phenotypic changes in adjacent medial cells^32^ and neuroinflammatory changes^37^ have been suggested.

This study has limitations. While our cohort of older human brains is larger than most previous studies, it remains relatively modest (n=52). This reflects our requirement for absent/minimal Alzheimer pathology, in the older brains studied. For the 2 experimental animal cohorts, we used only male animals so as to avoid potential confounding due to the female sex cycle. We have not prospectively tested a perturbation of vascular collagen-IV, as we currently lack experimental models that exhibit brain arterial pathology characteristic of SVD.

In conclusion, our data suggest that genetic risk of SVD due to *COL4A1-COL4A2* appears likely to result from insufficient rather than excessive arterial collagen-IV. It appears likely that types I and III, but not type IV, collagens contribute to arterial thickening in SVD, possibly as an injury response. In 3 different species, chronic hypertension did not associate with altered vascular collagen-IV.

## Article Information

### Acknowledgments

We gratefully thank human tissue donors and their families. We acknowledge the Oxford Brain Bank, supported by the Medical Research Council (G1000691), Brains for Dementia Research and the National Institute for Health and Care Research (NIHR) Oxford Biomedical Research Centre. The figure in the graphical abstract was prepared using Biorender software.

### Sources of Funding

Alzheimer’s Society (United Kingdom; PG146/151), ADDF (Ref 20140901), Alzheimer’s Research UK (PPG2014A-8), and Medical Research Council (MR/R005567/1) to Dr Hainsworth. National Institute for Health and Research Clinical Lectureship (CL-2015-16-001) to A.B. Shtaya. Wellcome Trust (204809/Z/16/Z) to Dr Meijles. National Institutes of Health (P01-NS40256-10, P50-AG16574-14, and P50-AG2571105) to Drs Dickson and Lin. National Institute on Aging (NIA)-funded Program Project (P01-AG000001) and National Institute of Neurological Disorders and Stroke (NINDS)-funded Program Project (P01-NS031649) to Dr Rosene. Italian Ministry of University and Education and “Dipartimenti di eccellenza 2018-2022” to Dr Zamboni. Stroke Association (16VAD-04), Heart Research UK (RG 2664/17/20) and Medical Research Council (MR/R005567/1) to Dr Van Agtmael. This research was funded, in part, by the Wellcome Trust (grant number 204809/Z/16/Z). For the purpose of open access, the author has applied a CC-BY public copyright licence to any Author Accepted Manuscript version arising from this submission.

### Disclosures

Dr Hainsworth has received honoraria from Eli-Lilly and NIA. He leads Dementias-Platform UK Vascular Experimental Medicine group. The other authors report no conflicts.

### Supplemental Material

Supplemental Methods

Table S1

Figures S1–S3

References 38,39

## Supplementary Material


