# Supplementary material for: Vascular Collagen Type-IV in Hypertension and Cerebral Small Vessel Disease
Source: Stroke. 2022 Oct 7;53(12):3696–705. doi: 10.1161/STROKEAHA.122.037761 (PMC9698121; doi:10.1161/STROKEAHA.122.037761)
Supplement: Supplementary file 1 [file str-53-3696-s001.pdf]

SUPPLEMENTAL MATERIAL

Vascular collagen-IV in hypertension and cerebral small vessel disease

Apoorva Kumar BSc[1,2], Natalie Yeo MBBS[1], Max Whittaker MBBS[1], Priya Attra MBBS[1], Thomas R Barrick PhD[1], Leslie R Bridges FRCPath[1,3], Dennis W Dickson MD[4], Margaret M Esiri FRCPath[5], Chad W Farris PhD[6], Delyth Graham PhD[7], Wen Lang Lin PhD[4], Daniel N Meijles PhD[1], Anthony C Pereira FRCP[1,2], Gregory Perry MSc[1], Douglas L Rosene PhD[6], Anan B Shtaya FRCS[1], Tom Van Agtmael PhD[7], Giovanna Zamboni DPhil[5,8], Atticus H Hainsworth PhD[1,2,]*

[1]Molecular and Clinical Sciences Research Institute, St George's, University of London, London, SW17 0RE, United Kingdom. [2]Neurology, [3]Cellular Pathology, St George's University Hospitals NHS Foundation Trust, Blackshaw Road, London, SW17 0QT, United Kingdom. [4]Department of Neuroscience, Mayo Clinic, 4500 San Pablo Road, Jacksonville, FL 32224, USA. [5]Nuffield Department of Clinical Neurosciences, Oxford University, John Radcliffe Hospital, Oxford, OX3 9DU, United Kingdom. [6]Department of Anatomy and Neurobiology, Boston University School of Medicine, 700 Albany Street, Boston, MA, 02118-2394, USA. [7]Institute of Cardiovascular & Medical Sciences, University of Glasgow, University Avenue, Glasgow, G12 8QQ, United Kingdom. [8]Dipartimento di Scienze Biomediche, Metaboliche e Neuroscienze, Università di Modena e Reggio Emilia, Via Giardini 1355, 41126 Modena, Italy.

*Correspondence: Dr Atticus H Hainsworth PhD, ORCID: orcid.org/0000-0001-7877-8013

St George's University of London, Cranmer Terrace, London, SW17 0RE, United Kingdom. Email: ahainsworth@sgul.ac.uk

Supplementary Materials and Methods

Human cohort

Human tissue samples were supplied by Oxford Brain Bank (REC approval#15/SC/0639). Ethical approval for this study was provided by NHS-Research Ethics Service (Ref#12/EM/0028). We selected all cases from the Oxford Brain Collection with minimal Alzheimer's disease neuropathology (Braak stage 0-II) for whom brain imaging data were available.

Human clinical measurements

Medical history, including a documented history of hypertension, the use of antihypertensive medication (never/former/current) were collected from subjects and checked with family doctors' computerized records. History of hypertension was defined as systolic blood pressure >140 mmHg, or diastolic blood pressure >90 mmHg, or use of anti-hypertensive medication.

Radiological assessment of white matter lesions

Brain CT scans were performed on Siemens DR or ART scanners (Siemens AG, Munich, Germany). Scans were performed 591 [250, 1521] days prior to death (median [IQR]; range: 51 – 4097 days). CT data for these cases have been previously reported[38, 39]. In the present study, scans were reviewed at window width 80/40 for supratentorial slices (10mm slices). The severity of white matter lesions was rated on a categorical scale, range: 0/1/2/3, based on previously published scales[21, 22]. A score of 0 indicated absence of hypodensity; 1 indicated questionable hypodensity, considered normal for age; 2 indicated periventricular hypodensity; 3 indicated marked hypodensity reaching the cortical grey matter. Scans were independently rated by an experienced neuroradiologist (Dr Rita Marasco) and by a neurologist with expertise in cerebrovascular disease (G.Z.). In the event of discordant ratings, cases were reviewed and consensus reached in all cases.

Neuropathological assessment of SVD

Assignment to neuropathological "SVD" was based on microscopic examination of haematoxylin and eosin sections by a registered neuropathologist (M.M.E. or Dr Catherine Joachim). Characteristics of the groups with and without SVD neuropathology are shown in Table 1. SVD was defined by vasculopathy-oriented criteria, as in our previous studies[2, 20]. These included: hyaline thickening of arteriolar walls; widened perivascular spaces; parenchymal changes considered to result from SVD (perivascular pallor of myelin staining, loosening with attenuation of nerve fibres with gliosis in white matter or loss of nerve cells and gliosis in deep grey matter) in one or more sections.

Human brain immunohistochemistry

A well-defined cohort of older individuals who had minimal AD pathology (Braak stage 0-II) were studied as in our previous report[20], see Table 1. Frontal and parietal cortical tissue blocks including subcortical white matter were examined. Formalin-fixed paraffin embedded sections were immunohistochemically labelled as described previously [20] using a monoclonal antibody selective for type IV collagen, specifically for α1(IV) and α2(IV) collagens (clone COL-94, mouse IgG1, Sigma-Aldrich, Poole, UK).

Paraffin wax embedded sections (6 µm thickness) were processed for immunohistochemistry as in our previous studies[20]. Endogenous peroxidase activity was blocked by exposure to H_2O_2 (3% v/v, aqueous solution) for 8 min. After high-pressure heat-induced antigen retrieval (30 s, 125 °C, in pH 7.8 Tris-citrate buffer), non-specific binding was blocked with phosphate buffered saline containing 0.1 % v/v Triton-X100 and 3 % (w/v) bovine serum albumin (PBT-BSA) for 60 min at room temperature. Sections were then exposed to primary antibodies at 4 °C overnight.

Primary antibodies were diluted on the day of use in PBT-BSA. Primary antibodies were collagen-IV (mouse monoclonal, clone COL-94, diluted 1:800; Sigma-Aldrich, Poole, UK), collagen-I (rabbit polyclonal, 1:1200; Abcam, Cambridge, UK), collagen-III (rabbit polyclonal, 1:2000; Rockland Immunochemicals, Limerick, PA). Antibody labelling was visualised using a peroxidase-conjugated secondary reagent and diaminobenzidine (DAB) chromagen (Envision® kit, K4065, Dako-Agilent, Ely, UK) then counterstained with Mayer's haematoxylin. As a negative control, neighbouring sections were treated identically with an irrelevant primary antibody (monoclonal against HPV, 1:800; Dako, Glostrup, Denmark).

Assessment of collagen-IV positive area fraction and sclerotic index

Sections were viewed on a Nikon Eclipse Ni-E Upright microscope with 20x or 40x objective lens. All vessels of arterial appearance within subcortical white matter in the size range 40 – 150 µm least outer diameter were digitally sampled as TIFF files. To estimate the collagen-IV positive area fraction of each vessel wall, TIFF files were opened in Fiji-ImageJ software (https://imagej.net/Fiji). The background and lumen contents were removed (an example is shown in Figure 1, in the main document). Each image underwent colour deconvolution using the H-DAB macro within FiJi software, then a threshold detection algorithm within FiJi software was applied, using the default filter, to identify all collagen-IV positive pixels within the vessel wall (Figure 1F, main document). Collagen-IV positive area fraction (AF, as %) was calculated as AF = 100 x (collagen-IV positive vessel wall area/total vessel wall area) for each vessel. Mean AF was calculated from all vessels meeting the inclusion criteria for each case.

Sclerotic index was estimated as a measure of vessel wall thickening, using the same TIFF files. On inspection, all small arteries with non-inflected circular cross section were selected. Vessels with squat ellipsoid cross section were also included, defined by greatest diameter less than 2x smallest diameter. For all selected vessels, the inner diameter and outer diameter were measured along the line of smallest inner diameter. Sclerotic index was computed as 1 - (inner diameter/outer diameter) and mean sclerotic index recorded for each case.

Harvesting of TIFF files and all image analyses were performed blind to clinical data.

Non-Human Primate model

Brain tissue from 17 male rhesus monkeys *Macaca mulatta* was used in this study[24]. This tissue had been collected and stored as part of a NIA-funded Program Project ("Neural Substrates of Cognitive Decline in Aging Monkeys," P01-AG000001) and a NINDS-funded Program Project ("Cognition and Cerebrovascular Disease," P01-NS031649). Monkeys were housed in the Laboratory Animal Science Center of Boston University, which is accredited by the Association for the Assessment and Accreditation of Laboratory Animal Care. All animals were treated with strict accordance to the standards of the NIH Guide for the Care

and Use of Laboratory Animals. Only male animals were studied to avoid confounding protective effects due to the female sex cycle. No unexpected mortality was seen in this cohort prior to planned sacrifice.

Archival brain tissue was used from adult male *M. mulatta* that had been randomised either to undergo aortic narrowing to induce chronic severe hypertension or to remain unoperated (normotensive controls). Chronic hypertension was produced by surgically coarcting the aorta, details of which have been previously described[24]. Briefly, a 1-cm segment of thoracic aorta, at a level distal to the origin of the left subclavian artery, was narrowed to a luminal diameter of 2.0–3.0 mm. The coarctation of the aorta resulted in a decrease in luminal area of about 80%, as indicated by autopsy findings. The normotensive animals were unoperated controls. Though formal sample size calculations were not conducted, group sizes were based on a priori determined outcomes and previous studies from the present group[24].

MRI scans were acquired using a Philips 3 Tesla scanner located in the Center for Biomedical Imaging at Boston University School of Medicine. Animals were initially anesthetized with ketamine hydrochloride (10 mg/kg, i.m.) and xylazine (1.25 mg/kg, i.m.) prior to MRI scanning. Supplemental doses of ketamine hydrochloride were then given as necessary to maintain a sufficient level of anesthesia.

Immunohistochemical Staining for Type IV Collagen. 30µm thick sections were immunolabelled with type IV collagen antibody (mouse monoclonal, clone COL-94, Sigma-Aldrich, St. Louis, MO) diluted 1:10,000 in potassium phosphate buffered saline (PPBS) for 48 h at 4 ^{o}C. Antibody labelling was detected using biotinylated goat anti-mouse secondary antibody (Vector Laboratories, Burlingame, CA) diluted 1:600 in PPBS, then avidin/biotinylated peroxidase complexes (Elite ABC Kit, Vector Laboratories, Burlingame, CA). Sections were counterstained with thionin to visualize cytoarchitecture.

Collagen-IV immunolabelled sections were scanned on a Nanozoomer slide scanner (Hamamatsu, UK) with 20x objective lens. Images were sampled as TIFF files in a stereotypical fashion from subcortical white matter areas. Tears, folds and other artefacts in the section were avoided. To estimate area fraction of collagen-IV immunoreactivity, TIFF files were imported into ImageJ software (http://rsb.info.nih.gov/ij/) and an automated threshold applied using a colour deconvolution plug-in. DAB-labelled area was determined, and collagen-IV-positive area fraction as a percent of the total area of the tiff file was calculated. Image analyses were performed blind to age or hypertensive status.

Hypertensive and normotensive rats

Rat studies were approved by the University of Glasgow Ethical Review Panel and complied with the Animals (Scientific Procedures) Act 1986 under Project Licence PPL-60/4286. Inbred colonies of the stroke-prone spontaneously hypertensive strain (SHRSP) and Wistar Kyoto (WKY) parent strain were maintained in the University of Glasgow by brother-sister mating. Maintenance of the colony integrity as well as the hypertensive and normotensive phenotypes was achieved by brother x sister mating, selecting SHRSP adult breeders with average systolic blood pressure 170-190 mmHg (males) and 130-150 mmHg (females), and WKY adult breeders with blood pressure <140mmHg (males) and <130mmHg (females) measured by tail-cuff plethysmography at age 12 weeks. Formal sample size calculations were not conducted, and group sizes were based on a priori determined outcomes and previous studies from the present group[19]. Only male animals were studied to avoid confounding protective effects due to the female sex cycle.

For this study male rats aged ≥10 months were deeply anaesthetised with 1.5-2.5 % isoflurane in a mixture of N_2O/O_2 (70/30) then heparinised saline was introduced transcardially, followed by 4 % paraformaldehyde (PFA) in phosphate-buffered saline (at least 200 ml, 80-100 mmHg perfusion pressure). Brains, while still in the skull, were post-fixed in 4% PFA for 24 hours then removed and post-fixed for a further 24 hours. The brains were then cut into coronal blocks of 5 mm thickness and wax-embedded under vacuum. Adjacent sections (10 µm) were stained with haematoxylin and eosin (H&E) or with Masson trichrome stain.

For immunohistochemical labelling sections were processed as in our previous report[19]. Sections underwent heat-induced antigen retrieval (120 ^{o}C, 0.5 min) and blocking for 60 minutes at room temperature with 3 % (w/v) bovine serum albumen in phosphate-buffered saline containing 0.1 % Triton-X100 (PBT-BSA). They were then exposed to primary antibody to collagen type IV (rabbit polyclonal; Rockland Immunochemicals, Gilbertsville, PA) diluted 1:300 in PBT-BSA overnight at 4 ^{o}C. Immunolabelling was visualised using a peroxidise-conjugated secondary reagent and diaminobenzidine (DAB) chromagen (Envision® kit, Dako, Ely, UK), then lightly counterstained with Mayer's haematoxylin. As negative controls, matched sections treated identically but with irrelevant primary antibody (rabbit polyclonal anti-sheep IgG).

Sections were examined on a Zeiss Axioplan-2 microscope driven by Axiovision software (version 4.7). To study blood vessel morphometry a single blinded observer (P.A.) sampled all intact large leptomeningeal vessels at the ventral surface of the brain adjacent to the optic tract in coronal sections 0.5-1.5 mm anterior to bregma. To determine sclerotic index (S.I.=1 – inner diameter/outer diameter) as a measure of vessel wall thickening, vessels with a non-inflected circular or squat elliptical cross-sectional profile were sampled. To estimate area fraction of collagen-IV immunoreactivity within leptomeningeal vessels, images sampled as tiff files were imported into ImageJ software (http://rsb.info.nih.gov/ij/) and an automated threshold applied using a colour deconvolution plug-in. Inner and outer borders of vessel profile were delineated using a cursor and wall cross-sectional area estimated. DAB-labelled area within the vessel wall was determined, and collagen-IV-positive area fraction calculated. Image analyses were performed blind to hypertensive status.

Immuno-electron microscopy.

Thin sections of human brain were immunogold labelled for collagens I, III and IV (Rockland Immunochemicals, Gilbertsville, PA) as in our previous work[23]. Human cerebral cortical tissue from the superior frontal gyrus was sampled from formalin-fixed brains (n=6, ages: 46, 49, 51, 55, 62 and 71 years). These were processed for routine electron microscopy and post embedding immunogold EM. Briefly, small pieces (approx.1 mm^3) of tissues were dehydrated in 30%, 50%, 70%, and 90% EtOH, infiltrated, and embedded in LR White resin (Polysciences, Warrington, PA). Ultrathin sections collected on Formvar-coated nickel grids were incubated in primary antibodies overnight at 4°C, followed by secondary antibodies conjugated with 10 nm colloidal gold particles (Jackson ImmunoResearch Laboratories, Inc, West Grove, PA, USA). Sections were stained briefly with uranyl acetate and lead citrate before examination with a Philips 208S electron microscope.

Table S1. Demographic data for females and males in the cohort of aged human brains

	All	Female	Male
N	52	22	30
Age in years, mean (SD)	82.8 (7.0)	86.6 (5.75)	79.9 (6.42)
Clinical history of hypertension. (data available for n=42)	18/42 (42.9%)	5/19 (26.3%)	13/23 (56.5%)
Presence of at least one *APOE4* allele	13/52 (25%)	6/22 (27%)	7/30 (23%)
Presence of neuropathological SVD defined according to Esiri et al. 1997.	26/52 (50%)	11/22 (50%)	15/30 (50%)
Radiological white matter lesion score (range: 0-3). Median, [IQR] (data available for n=44)	1 [0, 2]	1 [1, 2]	1 [0, 2]
Radiological lacunar lesion total. Median, [IQR]	0 [0, 2]	0 [0, 0.5]	1 [0, 2]
Neuropathological score for cerebral amyloid angiopathy (range 0-24). Median [IQR]	0 [0, 3]	0 [0, 3]	0 [0, 3]
Neuropathological history of haemorrhage	5/52 (9.6%)	2/22 (9.1%)	3/30 (10%)
Post mortem interval in hours. Median, [IQR]	47 [28, 74]	41 [24, 71.5]	51.5 [28, 74]

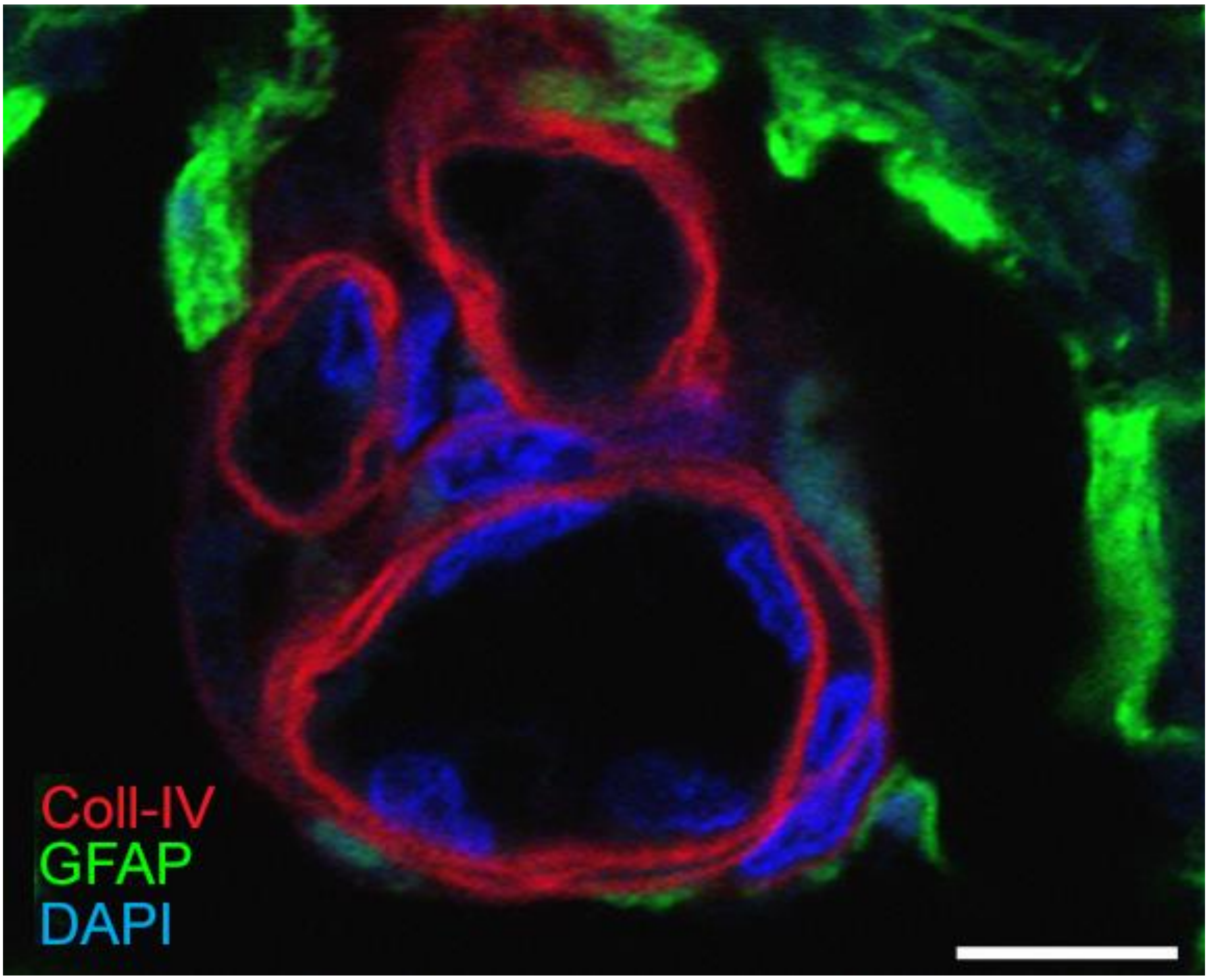

Figure S1. Confocal imaging confirms distinct layers of subendothelial and adventitial collagen-IV immunoreactivity.

Small penetrating artery in frontal subcortical white matter of an older person. Collagen-IV (red) reveals distinct subendothelial and adventitial layers. Astrocytic endfeet, labelled with GFAP (green), demarcate the boundaries of the perivascular space. Cell nuclei are visualised with the chromatin marker DAPI (blue). Bar: 10 microns.

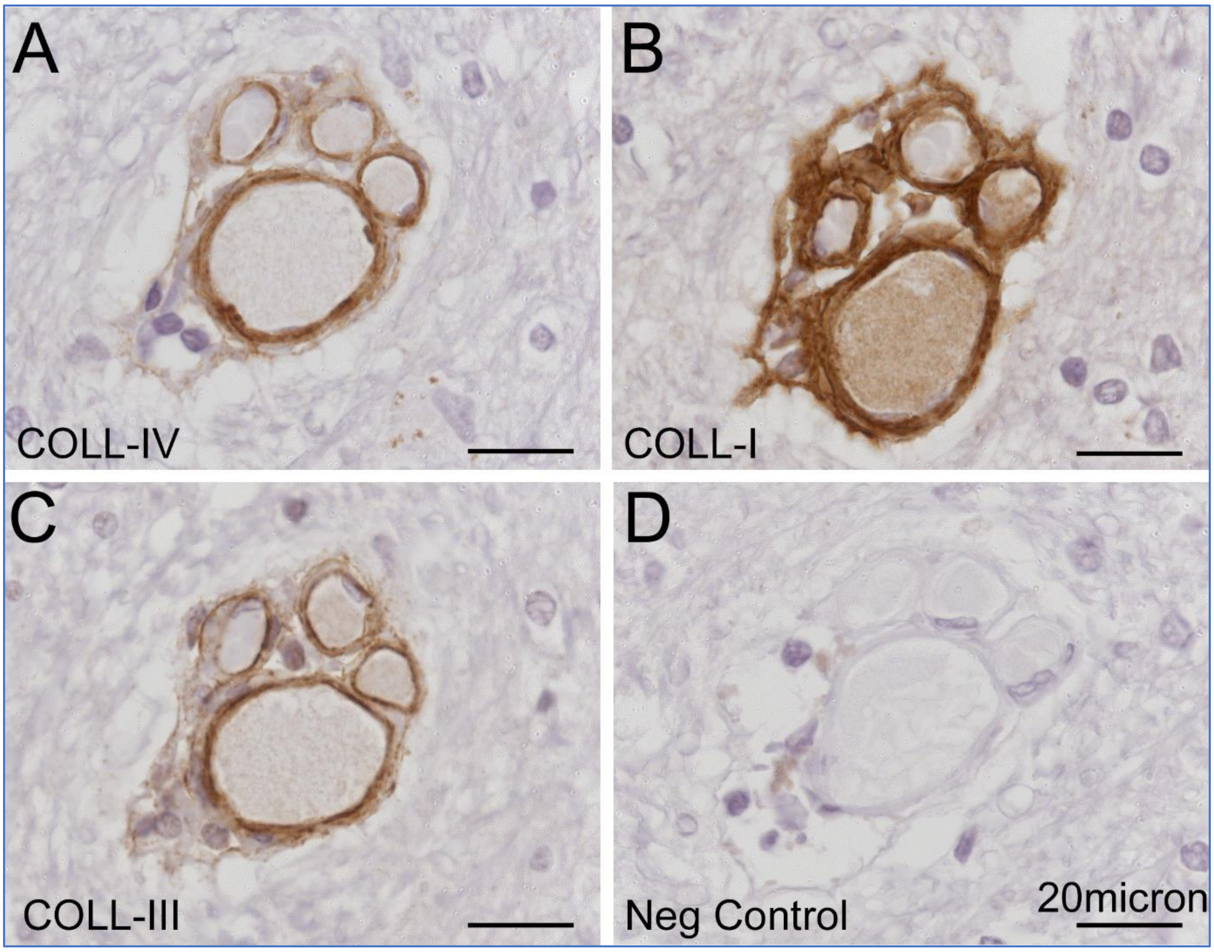

Figure S2. Collagen types I, III and IV in small vessel disease.

A small penetrating brain artery, in an older person with neuropathological diagnosis of SVD. A-C: immunolabelling for collagen type IV (panel A), type I (B) and III (C). D: negative control, a neighbouring section treated with an irrelevant antibody (monoclonal anti-HPV). Immunolabelling is visualised with DAB chromogen (brown). Haematoxylin nuclear counterstain (blue). Scale bars: 20 µm.

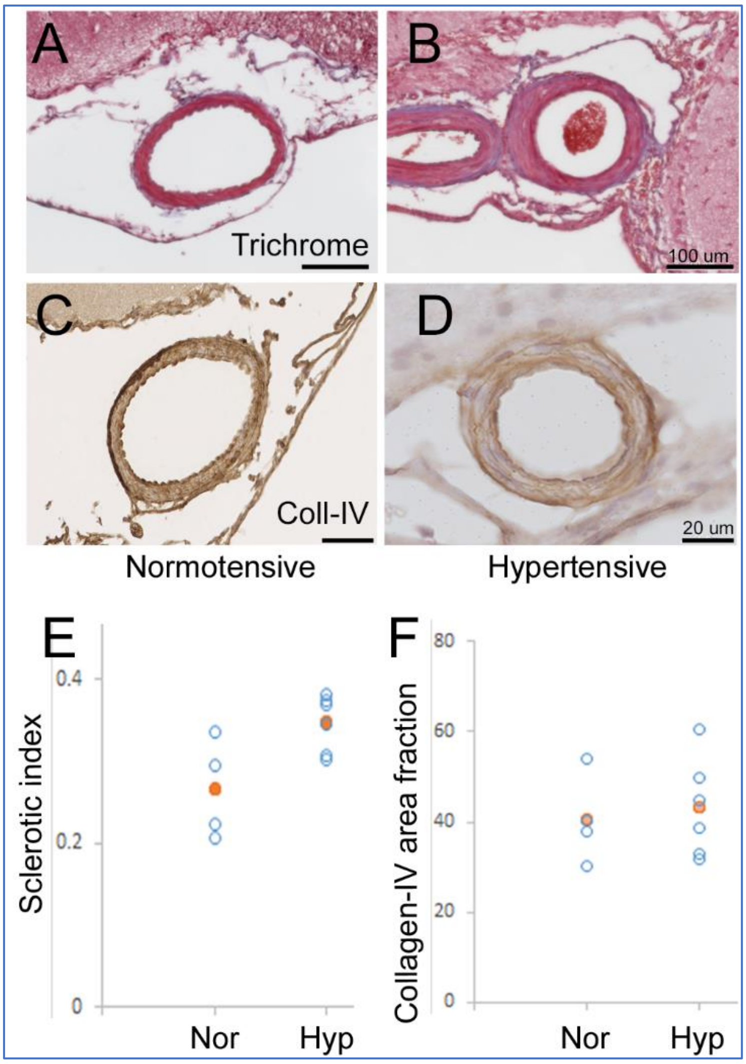

Figure S3. Cerebral arterial fibrosis in chronically hypertensive rats.

A-D: leptomeningeal arteries of chronically-hypertensive rats (male SHRSP strain, age 10-12 months) and normotensive age-matched male normotensive rats of the parent strain (Wistar-Kyoto, WKY). Relative to normotensive rats (panel A) the hypertensive animals exhibited pronounced fibrosis in the arterial vessel wall, seen with the blue trichrome stain (B). Collagen-IV immunolabelling (brown) was evident as part of extracellular matrix throughout the artery wall in normotensive (panel C) and hypertensive (D) rats. Scale bars: 100 µm (A, B), 20 µm (C, D).

E, F: sclerotic index (E) and extent of vascular collagen-IV immunolabelling (F) were quantified in leptomeningeal arteries from normotensive (Nor) WKY and hypertensive (Hyp) SHRSP rats (n=4, 7, respectively). Open blue symbols represent individual animals, filled orange symbols show group means. The arterial sclerotic index was higher in hypertensive SHRSP relative to normotensive WKY rats (P=0.038, Mann-Whitney U-test) in accord with our previous report{Brittain, 2013 #1239}. The extent of vascular collagen-IV immunolabelling in these vessels was not significantly different (P=0.762, Mann-Whitney U-test).

References cited in Supplementary File (numbers according to Main document)

2. Esiri MM, Wilcock GK, Morris JH. Neuropathological assessment of the lesions of significance in vascular dementia. *J Neurol. Neurosurg. Psychiatry*. 1997;63:749-753

19. Brittain JF, McCabe C, Khatun H, Kaushal N, Bridges LR, Holmes WM, et al. An MRI-histological study of white matter in stroke-free SHRSP. *J. Cereb. Blood Flow Metab*. 2013;33:760-763

20. Bridges LR, Andoh J, Lawrence AJ, Khoong CH, Poon WW, Esiri MM, et al. Blood-brain barrier dysfunction and cerebral small vessel disease (arteriolosclerosis) in brains of older people. *J. Neuropathol. Exp. Neurol*. 2014;73:1026-1033

21. Blennow K, Wallin A, Uhlemann C, Gottfries CG. White-matter lesions on CT in Alzheimer patients: relation to clinical symptomatology and vascular factors. *Acta Neurol Scand*. 1991;83:187-193

22. Scheltens P, Erkinjunti T, Leys D, Wahlund LO, Inzitari D, del Ser T, et al. White matter changes on CT and MRI: an overview of visual rating scales. European Task Force on Age-Related White Matter Changes. *Eur Neurol*. 1998;39:80-89

23. Lin WL, Wszolek ZK, Dickson DW. Hereditary diffuse leukoencephalopathy with spheroids: ultrastructural and immunoelectron microscopic studies. *Int J Clin Exp Pathol*. 2010;3:665-674

24. Moore TL, Killiany RJ, Rosene DL, Prusty S, Hollander W, Moss MB. Impairment of executive function induced by hypertension in the rhesus monkey (Macaca mulatta). *Behav. Neurosci*. 2002;116:387-396

38. Mendes Ribeiro HK, Barnetson LP, Hogervorst E, Molyneux AJ. A new visual rating scale for white matter low attenuation on CT. *Eur. Neurol*. 2001;45:140-144

39. Hogervorst E, Ribeiro HM, Molyneux A, Budge M, Smith AD. Plasma homocysteine levels, cerebrovascular risk factors, and cerebral white matter changes (leukoaraiosis) in patients with Alzheimer disease. *Arch. Neurol*. 2002;59:787-793
